# Vascular cell adhesion molecule 1 in patients with severe osteoarthritis of the hip: A prospective cross-sectional study

**DOI:** 10.1007/s00508-019-1497-2

**Published:** 2019-04-29

**Authors:** Bastian Oppl, Christian Datz, Ursula Huber-Schönauer, Emma Husar-Memmer, Wolfgang Brozek, Peter Zenz, Eva Gollob, Christian Wurnig, Alfred Engel, Klaus Klaushofer, Jochen Zwerina, Johann Bartko

**Affiliations:** 1 1st Medical Department, Hanusch Hospital, Ludwig Boltzmann Institute of Osteology at the Hanusch Hospital of WGKK and AUVA Trauma Centre Meidling, Heinrich Collin-Str. 30, 1140 Vienna, Austria; 2 Department of Internal Medicine, Hospital Oberndorf, Teaching Hospital of the Paracelsus Private Medical University of Salzburg, Oberndorf, Austria; 3 Department of Orthopedic Surgery, Otto Wagner Hospital and Karl Landsteiner Institute for Orthopedic Surgery, Vienna, Austria; 4 1st Orthopedic Department, Orthopedic Hospital Vienna Speising, Vienna, Austria; 5 Department of Orthopedics, SMZ Ost Donauspital, Vienna, Austria; 6 Institute of Rheumatology of the City of Baden, Baden, Austria

**Keywords:** CD106, Biomarkers/blood, Aged, Adult, Arthroplasty

## Abstract

**Background:**

Osteoarthritis (OA) of the hip is a frequent and debilitating joint disease. Only few clinical risk factors for hip OA are established and clinically applicable biomarkers to identify patients at risk are still lacking. The glycoprotein vascular cell adhesion molecule 1 (VCAM-1) is expressed by chondrocytes and synovial tissue and was a predictive marker for development of severe large joint OA in a previous study.

**Objective:**

It was tested whether increased serum levels of VCAM-1 are prevalent in patients with severe OA of the hips.

**Methods:**

In this prospective, multicenter, cross-sectional study, risk factors of severe hip OA were investigated in patients scheduled for hip joint arthroplasty and 100 patients were randomly selected for validation of VCAM-1 as a potential biomarker for hip OA. Serum samples were analyzed by an enzyme-linked immunosorbent assay and compared with a sex and age-matched control cohort.

**Results:**

The groups were similar in age, gender ratio and prevalence of diabetes. Serum concentrations of VCAM-1 were 8% higher in OA patients compared to controls, without reaching statistical significance (818 ng ml^−1^, 95% confidence interval, CI 746–891 ng ml^−1^ versus 759 ng m^−1^, 95% CI 711–807 ng ml^−1^; *P* = 0.4839).

**Conclusion:**

The results of this study show that serum concentrations of VCAM-1 cannot distinguish patients with severe hip OA from age and sex-matched controls.

## Introduction

Osteoarthritis (OA) is one of the leading causes of disability [1] and affects approximately 15% of the general population aged 15 years and older [2]. An estimated 25% of the adult population will suffer from OA by 2030 [3]. Apart from mechanical factors (e. g. hip dysplasia), little is known about risk factors for development of hip OA. For instance, higher body weight and the presence of metabolic syndrome components are commonly associated with knee OA [4, 5], but less frequently with hip OA [6–8]. Age is a consistent but non-modifiable risk factor [9].

Vascular cell adhesion molecule 1 (VCAM-1), a 110 kDa glycoprotein expressed by endothelial cells, mediates leukocyte adhesion during inflammation [10, 11]. The VCAM-1 is constitutively expressed on chondrocytes and synovial tissue. On stimulation by proinflammatory cytokines, VCAM-1 is strongly up-regulated [12, 13], suggesting that VCAM-1 mediates adhesion of immune cells in the inflamed joint. Serum VCAM-1 levels were predictive for large joint OA in a prospective study [14] and with knee OA progression in another study [15]. Thus, soluble VCAM-1 is proposed as a biomarker for risk prediction of future joint replacement [14, 16]. This study investigated whether serum levels of VCAM-1 are associated with the presence of severe hip OA and compared patients undergoing total hip joint replacement with an age and sex-matched control cohort.

## Methods

### Study population

In this prospective, multicenter, cross-sectional study, risk factors of severe OA were investigated in patients scheduled for hip joint arthroplasty (clinical trial registry www.drks.de, identifier: DRKS00003334) and 100 patients were randomly selected for validation of VCAM-1 as a potential biomarker for OA. The ethics committee of the City of Vienna reviewed and approved the study protocol. The study was conducted in accordance with the ethical standards laid down in the Declaration of Helsinki at four study sites: the Ludwig Boltzmann Institute of Osteology at Hanusch Hospital of WGKK and AUVA Trauma Centre Meidling, Vienna, the Orthopedic Hospital Vienna Speising, Department of Orthopedic Surgery, Otto Wagner Hospital, Vienna, Department of Orthopedics, SMZ Ost Donauspital, Vienna. All participants gave written informed consent before study inclusion. The inclusion criteria were: Caucasian males and females aged 18–69 years and scheduled for hip or ankle joint arthroplasty due to OA. The exclusion criteria were: age < 18 or > 69 years and bone fractures as a reason for arthroplasty. As predefined by the study protocol, validation of VCAM-1 as a potential biomarker was a prespecified secondary outcome. As a control cohort, 100 serum samples from a population-based prospective study including subjects without total joint replacement of a national preventive screening program for colorectal cancer were randomly selected [17]. Subjects were matched for age and gender.

### Laboratory investigations

Aliquots of serum samples were stored at −80 °C until assays were performed. Serum levels of VCAM-1 were measured by commercially available enzyme immunoassays in accordance to the manufacturers’ instructions (Human sVCAM-1 Platinum ELISA, Bender MedSystems GmbH, Vienna, Austria). The lower limits of quantification were 0.6 ng ml^−1^, the intra-assay coefficient of variation was 3.1% and the inter-assay coefficient of variation was 5.2%.

### Sample size and statistical analysis

The sample size calculation was based on experiences with studies using VCAM-1 in OA patients. In a previous study, serum concentrations of 944 ± 590 ng ml^−1^ (standard deviation) were measured [14]. This corresponds to a mean increase of 40% compared to patients without joint replacement surgery. It was calculated that a similar increase could be detected with *n* = 100 per group. Statistical comparisons between groups were performed by using the Mann-Whitney U-test for continuous variables and Fisher’s exact test for categorical data.

## Results

### Demographic characteristics of patients

The study sample included 100 patients undergoing total joint arthroplasty of the hip and 100 individuals from a national preventive screening program for colorectal cancer (Table 1). The gender ratio was 1:1 in both groups. The mean age was 58.9 ± 10.4 years in the OA cohort and 59.9 ± 9.9 years in the control cohort. The mean body mass index (BMI) was ~10% higher in the OA cohort compared with the control cohort (*P* = 0.0056). The prevalence of diabetes was 10% in the OA group and 8% in the control cohort.

**Table 1:** Characteristics of study cohorts

	Controls (*n* = 100)		OA patients (*n* = 100)		*P*-value
Female, *n* (%)	50	(50)	50	(50)	1.0000
Age, years	59.9	±9.9	58.9	±10.4	0.8468
BMI (kg · m^−2^)	26.4	±3.9	28.7	±5.6	**0.0056**
Diabetes, *n* (%)	8	(8)	10	(10)	0.8056

*BMI* body mass index; Values represent mean ± SD unless otherwise indicated

**Bold text** indicates a *P*-value less than 0.05

### Serum levels of VCAM-1

**Fig. 1:**
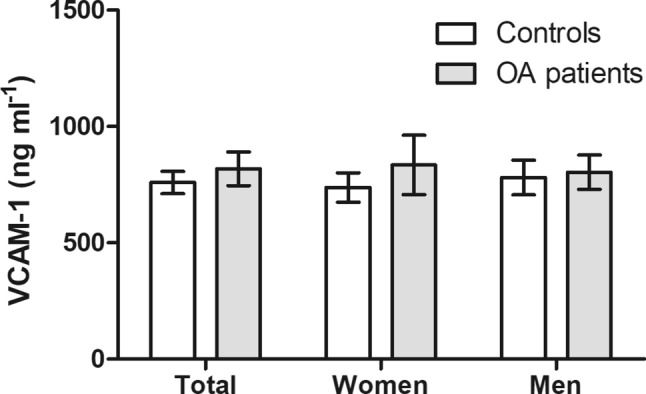
Serum levels of VCAM-1. Patients with severe OA (*n* = 100) had slightly higher (8%) VCAM-1 serum levels compared with an age and sex-matched control cohort from a preventive examination program (*n* = 100). This was more pronounced in women (+13%) and only marginally in men (+3%); however, all comparisons were not statistically significant. *VCAM-1* vascular cell adhesion molecule 1, *OA* osteoarthritis. Data are presented as means (95% CI)

Serum concentrations of VCAM-1 were 8% higher in OA patients compared with controls, but this was not significant (818 ng ml^−1^, 95% confidence interval, CI 746–891 ng ml^−1^ versus 759 ng ml^−1^, 95% CI 711–807 ng ml^−1^; *P* = 0.4839; Fig. 1). The differences were more pronounced in women (+13%; 834 ng ml^−1^, 95% CI 706–961 ng ml^−1^ versus 738 ng ml^−1^, 95% CI 675–801 ng ml^−1^; *P* = 0.5059) than in men (+3%; 803 ng ml^−1^, 95% CI 729–877 ng ml^−1^ versus 780 ng ml^−1^, 95% CI 706–854 ng ml^−1^; *P* = 0.7907). In both groups serum concentrations of VCAM-1 were not significantly correlated with BMI (OA patients r = 0.14, *P* = 0.1673; controls r = 0.10, *P* = 0.3343).

## Discussion

Osteoarthritis of the hip is a common condition in the aging population. To date, there are no specific biochemical markers for diagnosis, prediction and outcome. As serum levels of VCAM-1 were shown to be predictive for the development of severe large joint OA [14], VCAM-1 may potentially be useful for clinical assessment. This prospective study evaluated whether serum levels of VCAM-1 can be associated with the presence of severe OA. Hip joint OA is associated with degradation of articular cartilage and extracellular matrix, but signs of inflammation may also be present in various degrees over time. The inflammatory activity in OA is of low grade, characterized by moderately increased levels of proinflammatory cytokines and proteases, which finally leads to matrix degradation [18]. The VCAM-1, a sialoglycoprotein of the immunoglobulin superfamily [19], is a driver of leukocyte adhesion to the vascular endothelium. In OA, VCAM-1 is up-regulated in the synovium and is also induced in chondrocytes on cytokine stimulation in vitro [12, 13]. This suggests that increased circulating levels of VCAM-1 are possibly derived from affected joints. In support of this hypothesis, a study in patients with rheumatoid arthritis showed that the levels of VCAM-1 were significantly higher in synovial fluid samples compared with simultaneously obtained plasma samples [22], indicating a translocation of soluble VCAM-1 from the joints to the systemic circulation; however, it is also possible that soluble VCAM-1 is derived from microvessels of the subchondral bone or the synovium.

In a longitudinal study, serum levels of VCAM-1 predicted joint replacement due to hip and knee OA, independent of age, sex, and BMI, over the course of 15 years [14]. Serum VCAM-1 levels of patients who developed OA were 40% higher at baseline. The association of high VCAM-1 serum levels with OA does not appear to be restricted to patients with knee or hip OA. Patients with erosive hand osteoarthritis were also reported to have 30% higher VCAM-1 concentrations in serum than controls [20]. Another study found serum levels of VCAM-1 to be associated with the number of affected joints in hand OA [21]; however, some conflicting data have also been reported. More consistent with the present results, a large population-based study investigated the link between markers of atherosclerosis and knee OA [15]. Interestingly, the study found an association of VCAM-1 with the presence of knee OA only among women. The lack of higher levels of VCAM-1 in the present study cohort compared to controls may have several reasons. Most studies investigated patients with knee or knee and hip arthropathy [14, 15, 22, 23], while the present study included patients who underwent total hip arthroplasty only. Another possible factor may include the use of nonsteroidal anti-inflammatory drugs (NSAIDs), such as ibuprofen and diclofenac, which were demonstrated to inhibit the expression of VCAM-1 directly on endothelial cells and generally be anti-inflammatory [24, 25]. Since many patients receive NSAIDs prior to total joint replacement [26, 27], it is likely that the present study cohort received NSAIDs on a regular basis, possibly contributing to lower levels of soluble VCAM-1. Thirdly, the control cohort only excluded patients with severe OA and joint replacement surgery. Thus, milder or underdiagnosed patients with OA may have confounded serum VCAM-1 levels; however, when considering VCAM-1 for clinical use, for example for discriminating diseased from non-diseased patients, comparing patients with a control group from a preventive examination program may be more generalizable and pragmatic. Highly selected cohorts such as healthy blood donors do not necessarily represent a suitable control group compared with persons in the general population (the healthy-volunteer effect) [28]. Another observation was that despite matching for age and sex, the mean BMI was significantly higher in the OA cohort. This is in accordance with previous studies, showing moderate evidence for a positive association between hip OA and obesity [6, 29]. Interestingly, VCAM-1 has also been implicated as a mediator of endothelial dysfunction in obesity [30]. Some studies observed higher VCAM-1 levels among individuals with higher BMI [22, 31], whereas others found no correlations [32, 33]. Similar to the latter, no significant correlation between VCAM-1 and BMI was found in this study sample, neither in OA patients nor in the control group. Overall, the results of the study indicate that the mean serum concentrations of VCAM-1 substantially overlap between patients with severe hip OA and age and sex-matched controls.
